# Editorial: Teratogenesis: Experimental Models, Mechanisms and Clinical Findings in Humans

**DOI:** 10.3389/fgene.2022.901400

**Published:** 2022-05-09

**Authors:** Lucas Rosa Fraga, Fernanda Sales Luiz Vianna, Miguel Del Campo, Maria Teresa Vieira Sanseverino, Lavínia Schuler-Faccini

**Affiliations:** ^1^ Laboratory of Genomic Medicine, Experimental Research Center, Hospital de Clínicas de Porto Alegre, Porto Alegre, Brazil; ^2^ Teratogen Information Service, Hospital de Clínicas de Porto Alegre, Porto Alegre, Brazil; ^3^ Department of Morphological Sciences, Institute of Health Sciences, Universidade Federal do Rio Grande do Sul, Porto Alegre, Brazil; ^4^ Graduate Program in Medicine, Medical Sciences, Universidade Federal do Rio Grande do Sul, Porto Alegre, Brazil; ^5^ Department of Genetics, Institute of Biosciences, Universidade Federal do Rio Grande do Sul, Porto Alegre, Brazil; ^6^ Graduate Program in Genetics and Molecular Biology, Universidade Federal do Rio Grande do Sul, Porto Alegre, Brazil; ^7^ Department of Pediatrics/Rady Children’s Hospital San Diego, School of Medicine, University of California San Diego, San Diego, CA, United States; ^8^ Pontifícia Universidade Católica do Rio Grande do Sul, PUCRS, Porto Alegre, Brazil

**Keywords:** congenital anomalies, thalidomide, zika virus, animal models, neural tube defects, bioinformatics, genetic susceptibility, gene-environment interaction

Congenital anomalies, also known as birth defects, are alterations that occur during intrauterine life that affect body structure or function. They can be identified prenatally, at birth, or even later in infancy. They have been identified in around 3–6% of births and are one of the main causes of neonatal and infant mortality worldwide (WHO, 2022). It is estimated that around 10% of congenital anomalies are caused by environmental factors, known as teratogens (Brent, 2009), but up to 25% are of multifactorial etiology, meaning that both genetic and environmental factors act in their development. Teratogens can be chemicals, which include drugs, biological agents (such as viruses and bacteria), and maternal conditions (for instance, diabetes and epilepsy), among others. Therefore, environmental exposures being potentially modifiable risk factors for congenital anomalies, represent an important opportunity to establish prevention strategies.

Studies in teratogenesis became prominent after thalidomide had been discovered as a teratogen. Since the 1960s, studies have been conducted to not only prevent birth defects, but also to quickly identify a teratogen, describe the malformative phenotypes associated with them, and investigate mechanisms related to these exposures. Indeed, several teratogenic agents have been identified since then through different clinical and epidemiological works; however, for most of these teratogens, the mechanisms of teratogenesis are not fully known. In this context, both clinical description and animal models are essential for the comprehension of such mechanisms, being useful for the evaluation of potential new teratogens and the development of preventive strategies. In addition, studies focusing on gene-environment interaction have been frequently used to bring insights regarding resistance or susceptibility to birth defects, as well as to identify phenotype modifiers related to the same teratogen. Currently, advances in molecular biology and bioinformatics studies allow us to better understand the molecular aspects involved in the processes of teratogenesis and similar pathways of genes and proteins unbalanced in a given exposure.

This research topic on *Teratogenesis: Experimental Models, Mechanisms and Clinical Findings in Humans* is a collection of six submissions that represent different arms of teratology. The article subjects range from topics on animal models to study teratogenesis and its mechanisms, bioinformatics, molecular aspects of teratogenesis as well as reviews on specific human birth defects related to environmental exposure.

The collection includes two reviews on animal models. As for most experimental studies involving animals, rodents are the most popular in studies in teratology. Interestingly, reviews on this research topic approach alternative species: non-human primates and chicken embryos. Li et al. present an interesting review on how non-human primates have contributed to research on teratogenesis, fetal and neonatal injury, and other adverse outcomes related to infections during pregnancy. They also bring some particular examples of pathogens that have been studied in non-human primates and discuss preventive measures for teratogenesis. Waccholz et al. review an alternative model for mammals, the chicken embryo. In their article, they focus on how the chicken embryo has contributed to the field of teratology and, since this model is not very widespread, they start with a review and historical aspects of it and then give examples of how it is used to evaluate phenotypes and mechanisms of teratogenic agents.

We then present papers focusing on an understanding of the mechanisms that are behind the teratogenic process to advance and deepen knowledge in teratology. Gomes et al. performed a systematic review focusing on genetic susceptibility to drug teratogenesis, investigating different teratogens and how certain genetic variants impact the risk for drug related-congenital anomalies. This systematic review included 29 studies, and a range of drugs and called attention to a gap in this type of study in the field of teratogenesis. Continuing this theme, Kowalski et al. used different computational biology approaches to investigate the teratogenic mechanisms of thalidomide. In this study, they performed a comparative analysis of proteins involved in known mechanisms of thalidomide teratogenesis to evaluate the interspecific phenotypic variability. After running several analyses and comparative genomics, they found many differences between animals affected and unaffected (less sensitive) to thalidomide teratogenicity, suggesting the need for further investigation of the molecular mechanisms involved with thalidomide through experimental studies.

The last two studies in this research topic approach the importance of careful clinical investigation associated with data from studies on molecular mechanisms in humans and experimental models to understand the etiology of birth defects and teratogenicity. In Finell et al., the different classifications and etiologies of neural tube defects are discussed. In this review article, the authors present the main causes of this group of congenital malformations, including maternal nutritional status, genetic risk factors, and teratogenic agents. They also discuss and give examples of these etiologic factors and how they interact. Finally, Schuler-Faccini et al. close this Research Topic with a review on the most recently discovered human teratogen, Zika virus. In their review, clinical aspects of children with Congenital Zika Syndrome and how prenatal exposure to the virus affects neurodevelopment are presented in detail. In addition, they discuss mechanisms of teratogenesis from experimental studies as well as cofactors related to the development of Congenital Zika Syndrome.

Overall, teratogenesis is a field that requires high epidemiological vigilance, accurate clinical description, as well as a combination of studies in human genetics and experimental studies. There are many human teratogens in which the mechanisms are poorly described or understood. Thus, the goal of this research topic is to provide studies aiming to describe clinical and genetic findings in humans and experimental studies in animal models and cells, which can increase knowledge of the mechanisms of human teratogens. This knowledge may help in health strategies to prevent the occurrence of some congenital anomalies.
